# Physical Fitness Percentiles of German Children Aged 9–12 Years: Findings from a Longitudinal Study

**DOI:** 10.1371/journal.pone.0142393

**Published:** 2015-11-06

**Authors:** Kathleen Golle, Thomas Muehlbauer, Ditmar Wick, Urs Granacher

**Affiliations:** 1 Division of Training and Movement Sciences, Research Focus Cognition Sciences, University of Potsdam, Potsdam, Germany; 2 University of Applied Science in Sport and Management, Potsdam, Germany; Institute of Preventive Medicine, DENMARK

## Abstract

**Background:**

Generating percentile values is helpful for the identification of children with specific fitness characteristics (i.e., low or high fitness level) to set appropriate fitness goals (i.e., fitness/health promotion and/or long-term youth athlete development). Thus, the aim of this longitudinal study was to assess physical fitness development in healthy children aged 9–12 years and to compute sex- and age-specific percentile values.

**Methods:**

Two-hundred and forty children (88 girls, 152 boys) participated in this study and were tested for their physical fitness. Physical fitness was assessed using the 50-m sprint test (i.e., speed), the 1-kg ball push test, the triple hop test (i.e., upper- and lower- extremity muscular power), the stand-and-reach test (i.e., flexibility), the star run test (i.e., agility), and the 9-min run test (i.e., endurance). Age- and sex-specific percentile values (i.e., P_10_ to P_90_) were generated using the Lambda, Mu, and Sigma method. Adjusted (for change in body weight, height, and baseline performance) age- and sex-differences as well as the interactions thereof were expressed by calculating effect sizes (Cohen’s *d*).

**Results:**

Significant main effects of Age were detected for all physical fitness tests (*d* = 0.40–1.34), whereas significant main effects of Sex were found for upper-extremity muscular power (*d* = 0.55), flexibility (*d* = 0.81), agility (*d* = 0.44), and endurance (*d* = 0.32) only. Further, significant Sex by Age interactions were observed for upper-extremity muscular power (*d* = 0.36), flexibility (*d* = 0.61), and agility (*d* = 0.27) in favor of girls. Both, linear and curvilinear shaped curves were found for percentile values across the fitness tests. Accelerated (curvilinear) improvements were observed for upper-extremity muscular power (boys: 10–11 yrs; girls: 9–11 yrs), agility (boys: 9–10 yrs; girls: 9–11 yrs), and endurance (boys: 9–10 yrs; girls: 9–10 yrs). Tabulated percentiles for the 9-min run test indicated that running distances between 1,407–1,507 m, 1,479–1,597 m, 1,423–1,654 m, and 1,433–1,666 m in 9- to 12-year-old boys and 1,262–1,362 m, 1,329–1,434 m, 1,392–1,501 m, and 1,415–1,526 m in 9- to 12-year-old girls correspond to a “medium” fitness level (i.e., P_40_ to P_60_) in this population.

**Conclusions:**

The observed differences in physical fitness development between boys and girls illustrate that age- and sex-specific maturational processes might have an impact on the fitness status of healthy children. Our statistical analyses revealed linear (e.g., lower-extremity muscular power) and curvilinear (e.g., agility) models of fitness improvement with age which is indicative of timed and capacity-specific fitness development pattern during childhood. Lastly, the provided age- and sex-specific percentile values can be used by coaches for talent identification and by teachers for rating/grading of children’s motor performance.

## Introduction

Children’s health and well-being is highly correlated with their physical fitness. Recently published studies [1–4] indicate that low levels of physical fitness (e.g., cardiorespiratory fitness, muscular endurance/power) are associated with an elevated risk of developing adverse physiological events (e.g., unbalanced body mass index, waist circumference, systolic blood pressure, plasma glucose, lipoprotein cholesterol, insulin resistance) in school-aged students.

Physical fitness is usually determined in school-aged children using health-related physical fitness batteries (i.e., field tests). Compared to more sophisticated laboratory-based test equipment, field tests are easy-to-administer, involve minimal equipment and personnel, demonstrate good validity and reliability [5], and a large number of subjects can be tested in a relatively small amount of time. Normative data derived from field tests have previously been used to identify subjects for health/talent promotion or to provide current objective recommendations for the assessment of physical fitness during physical education.

A number of studies provided percentile values in children and detected significant better performances for older as compared to younger children in nearly all physical fitness tests [6–10]. The same studies likewise observed sex differences for proxies of endurance, isometric voluntary muscular strength, muscular power, muscular endurance, speed, and agility [6, 7, 9–12]. In fact, better performances in measures of endurance (e.g., 20-m shuttle run), isometric muscular strength (e.g., handgrip strength), muscular power (e.g., standing broad jump, throw ball), muscular endurance (e.g., bent arm hang, sit-ups), speed (e.g., 50-yard dash), and agility (e.g., 4 x 10-m shuttle run) were reported in boys as compared to girls [6, 7, 10, 12]. However, girls showed better performances in flexibility tests (e.g., sit-and-reach) [7, 10, 12].

Findings from these studies are helpful for the identification of individuals with specific physical fitness characteristics (e.g., talent identification) and the quantification of performance differences between ages and sex. However, the above mentioned studies are methodologically flawed due to their cross-sectional nature. More precisely, percentile values were computed using a cross-sectional approach (i.e., between-subject comparisons of different age groups). This is a major limitation because such an approach does not allow to deduce true physical fitness development within subjects (i.e., individual changes in timing and tempo of growth and maturation) over time. In fact, Andersen and colleagues [13] examined 8-year-old children during a period of four years for their cardiorespiratory fitness (i.e., oxygen uptake during bicycle ergometry). The longitudinal data was compared with cross-sectional data obtained from 8- to 14-year-olds. The authors observed considerably higher fitness levels in children that were longitudinally followed as opposed to those children who were assessed in a cross-sectional analysis.

Based on these findings, it is essential to use longitudinal data of the same individuals if the goal is to provide percentile values and to determine age- and sex-related differences in fitness development. Thus, the aim of the present study was to longitudinally assess physical fitness in a large sample of 268 healthy boys and girls from age 9 to 12 years. More specifically, age- and sex-specific differences in physical fitness (i.e., agility, endurance, flexibility, muscular power, speed) were quantified and percentile values computed. It is hypothesized that physical fitness improves from age 9–12 and that sex-specific differences occur over time.

## Methods

### Sample and study design

**Fig 1:**
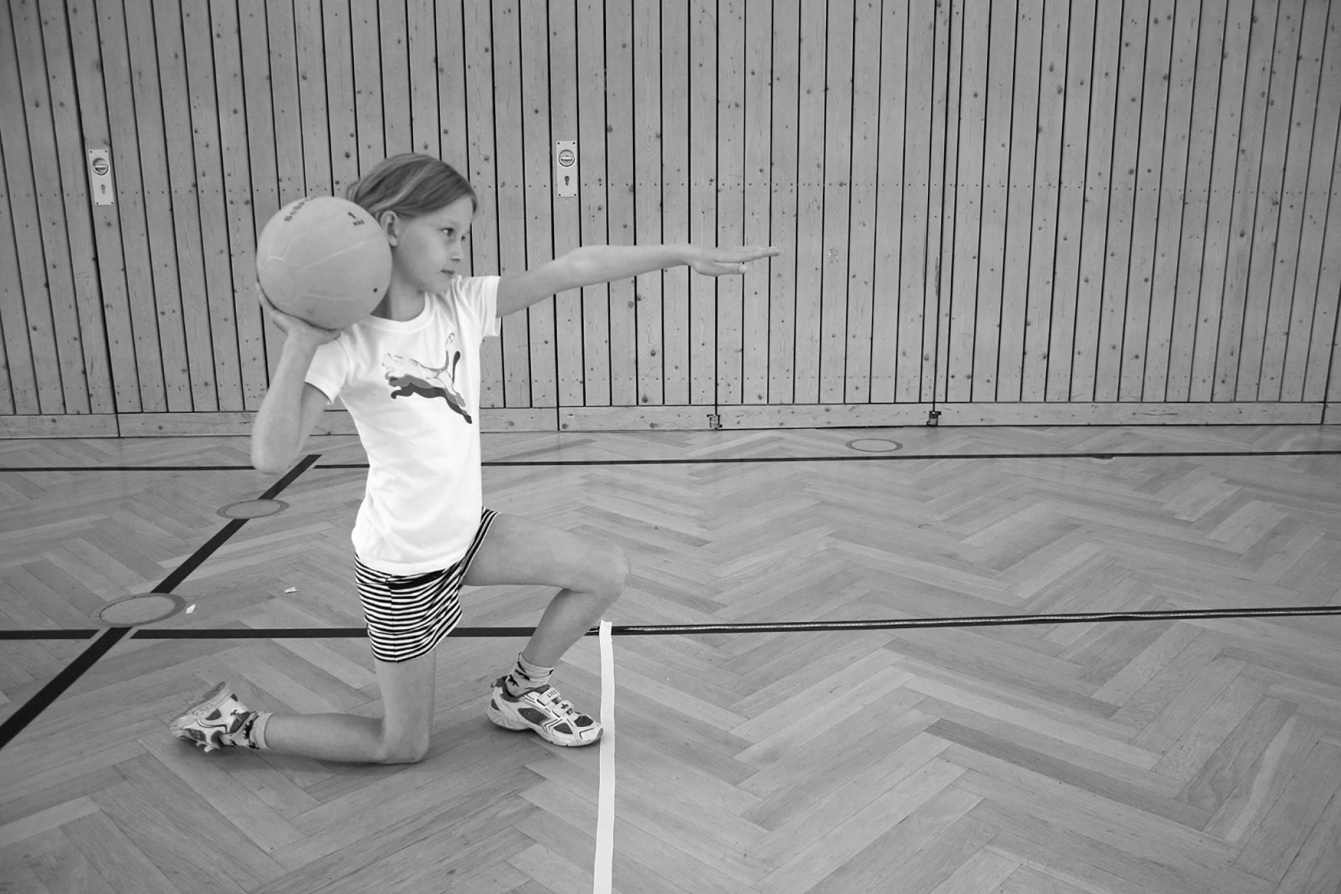
Schematic description of the ball push test. Note. The individual that is shown has given written informed consent (as outlined in PLoS ONE consent form) to publish these case details.

A longitudinal approach was conducted from 2006 to 2009 to test changes in physical fitness in 9- to 12-year-old children over time (i.e., from classes 3 to 6). The participating children attended 27 public primary schools that were randomly selected from urban (i.e., cities > 10,000 inhabitants) and rural (i.e., cities/villages ≤ 10,000 inhabitants) areas of the federal state Brandenburg (Germany) [14]. Each of the four testing periods over the four year study period lasted four weeks (always from March to April). The study was approved by the Ministry of Education, Youth and Sport of the federal state Brandenburg. Parents or legal representatives of each child provided written informed consent that included information regarding child´s birthdate. In addition, the individual that is shown in Fig 1 has given written informed consent (as outlined in PLoS ONE consent form) to publish these case details. The study was conducted according to the latest version of the declaration of Helsinki.

Four hundred and seventy students who attended grade three at the start of the study were invited to participate in the study. Informed consent and valid data were obtained from 364 children (146 girls, 218 boys) over the four year period. Chronological age with two decimals was calculated for each child as the difference between test date and birthdate. Age groups were categorized and classified as a whole year (i.e., 9.00–9.99 years ≙ 9 years, etc.). The age range of the enrolled children at the baseline tests (i.e., grade three) spanned from seven years (one girl), over eight years (48 girls; 53 boys), nine years (88 girls; 152 boys), ten years (nine girls; ten boys) up to eleven years (three boys). Due to the fact that computing percentile values in a longitudinal approach is limited to a single age group, only children aged nine (88 girls; 152 boys) were considered for further analysis. This same cohort was followed over the four year period and the children were tested in class 3 and in classes 4, 5, and 6.

### Anthropometry

Prior to physical fitness testing, body height was measured without shoes to the nearest 0.5 cm with a wall-mounted stadiometer (Seca, Basel, Switzerland). In addition, body weight was determined in light clothing and without shoes to the nearest 100 g with an electronic scale (Bodymaster vision BM-210, Rowenta, France). Body mass index (BMI) was calculated using body weight divided by height squared (kg/m^2^).

### Physical fitness tests

Physical fitness was determined using six different tests from motor fitness test batteries of Bös [15] and Stark [16]. The tests includeed the following items: 50-m sprint test (speed), 1-kg ball push test (upper-extremity muscular power), triple hop test (lower-extremity muscular power), stand-and-reach test (flexibility), star agility run test (agility), and 9-min run test (endurance). In accordance with Caspersen et al. [17], the applied physical fitness tests can be classified in health- (e.g., cardiorespiratory endurance, flexibility etc.) and skill-related (e.g., agility, speed, power etc.) components of physical fitness.

All tests were performed in the respective school gyms (except the 9-min run test) during official physical education classes using standardized test protocols. The physical fitness tests were conducted by qualified personnel (ensured by means of frequently conducted instruction classes) that hardly changed over the four year study period (i.e., testing in classes 3, 4, 5, 6). Qualification of the personnel was ensured by means of frequently conducted instruction classes. Furthermore, figures and illustrations were used to explain important characteristics for each test (for an example see Fig 1). A counter-balanced sequence of measurements was applied. Before testing, all students conducted a 10-minutes standardized warm-up program consisting of light running followed by different conditioning activities (e.g., side steps, backwards run, skipping, submaximal plyometric exercises, and short distance sprints).

#### 50-m sprint test

Speed performance was assessed in a stationary starting position [16]. Participants were instructed to stand in frontal erect posture with one foot right behind the starting line. Students started the first of two trials on the command ‘ready-set-go’ and accelerated at maximum effort. Time was taken with a stop watch to the nearest 1/10 s. The best trial (i.e., least running time) was used for further data analysis. The 50-m sprint proved to be reliable with an intraclass correlation coefficient (ICC) of 0.88 for the assessment of speed in 10- to 11-years-olds [18]. Further, the 50-m sprint is a valid test (*r* = 0.74 to 0.96) as compared to 100-m run performance in youth [19].

#### Ball push test

Muscular power of the upper extremities was assessed using the 1-kg medicine ball push test (Fig 1) [16]. The test was performed with the single left and right arm. For the right-handed push, participants set the right knee on the floor with a 90° angle between lower leg and thigh whereas the foot of the left leg stood behind the starting line with a 90° angle between lower leg and thigh of the left leg. The right hand held the ball at the neck. The extended left arm pointed forward at eye level (i.e., pushing direction). For the left-handed push the starting position for arms and legs changed, respectively (i.e., right arm pointed forward, left knee on the floor, and right foot at the starting line). In this frontal erect position, participants had to counter-rotate the trunk and push the ball as far as possible. The ball pushing distance was taken using a measuring tape to the nearest 25 centimeters (i.e., quarter of meter). Two trials were performed for each arm with a one minute rest before changing the pushing arm. The best trial in terms of maximal distance for each arm was summed and used for further data analysis. The ball push test is a reliable test (*r* = 0.82) for the assessment of upper-extremity muscular power in 8- to 10-years-olds [20].

#### Triple hop test

Lower-extremity muscular power was tested using the triple hop test [16]. Participants were instructed to stand with one foot right behind the starting line and to jump three times with the same leg as far as possible and to land on both feet. Subjects were allowed to use arm swing during the tests. Two trials were performed for each leg with a one minute rest between trials. The best trial in terms of maximal distance from the starting line to the landing point at heel contact for each leg was added and used for statistical analysis. Measurements were taken to the nearest centimeter using a tape measure. The triple hop test is a reliable test (*r* = 0.91) for the assessment of lower-extremity muscular power 12- to 14-years-olds [19]. The test is also valid (*r* = 0.74 to 0.96) when compared with the standing long jump test in youth [19].

#### Stand-and-reach test

Flexibility was tested using the stand-and-reach test [21]. Subjects were instructed to begin the test in a barefoot standing position on an elevated platform with feet together. They were asked to bend over using their maximal range of motion during expiration. During the test, knees, arms, and fingers were fully extended for at least two seconds. A tape measure was attached to the platform with 100 cm corresponding to the upper level of the platform. Values above 100 cm indicate that the person was able to reach beyond the toes (i.e., good flexibility). Values below 100 cm indicate that the person was not able to reach the toes (i.e., limited flexibility). The best out of two trials (i.e., maximal reach distance) with a one minute rest between trials was used for further data analysis. The stand-and-reach test is a reliable test (*r* = 0.94) for the assessment of flexibility in 7- to 11-years-olds [21].

#### Star agility run test

**Fig 2:**
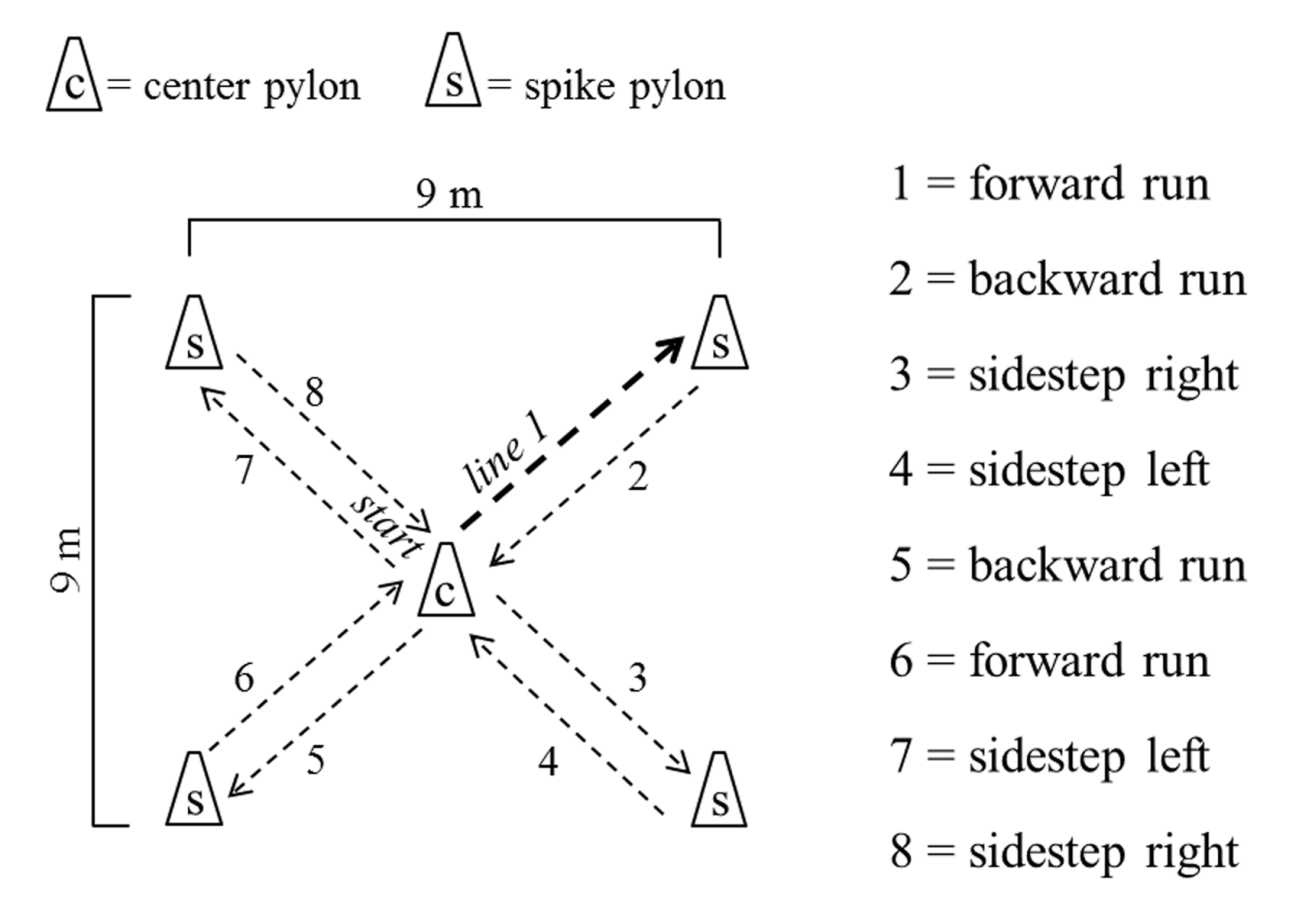
Schematic description of the star agility run test.

Agility was tested using the star agility run test [16]. Participants were instructed to run in different running techniques (e.g., forward, backward, side steps) from the center to the edge and back of a 9 x 9-m star-shaped field with four spikes (Fig 2). The spikes and the center of the field were each marked with pylons (height: 30 cm). Starting at the center of the field, the participants had to run forward to spike 1 (line 1) and backward to the center (line 2). From the center, they turned to the right side and side-stepped to spike 2 (line 3), turned to the left side and side-stepped back to the center (line 4). Upon reaching the center, students turned backward and ran to spike 3 (line 5) and forward to the center (line 6). Finally, they turned to the left side and side-stepped to spike 4 (line 7), turned to the right side and side-stepped back to the center (line 8). During the test, subjects had to touch the top of the pylons at each respective spike and when traversing the center position. Time was taken with a stop watch to the nearest 1/10 of a second. Subjects performed one practice trial and thereafter two test trials with a five minute rest in between. The best trial (i.e., least running time) was used for further data analysis. The star agility run test proved to be reliable with an ICC of 0.68 in 8- to 10-years-olds olds [20].

#### 9-min run test

The 9-min run test is a test for the assessment of aerobic capacity [16] and was performed outdoors. The 9-min run affords subjects to run the furthest distance during the nine minutes test time on a 400 m circuit athletic track. Participants started their run on the command ‘ready-set-go’. Split time was given every minute. The maximal distance achieved during the nine minute run was used for further data analysis. Excellent reliability has been reported for the 9-min run test with an ICC of 0.83 in 6- to 10-year-olds [22]. Validity of the 9-min run test was established in 10- to 12-year-olds using maximum oxygen uptake as gold standard and revealed significant correlation coefficients between running distance and VO2max in the range of r = 0.71–0.82 [23].

### Statistical analyses

Anthropometric and physical fitness test data were grouped by sex and age. Mean values and standard deviations were calculated for each group. Development of anthropometry and physical fitness over time was analyzed using a 2 (Sex: boys, girls) x 4 (Age: 9, 10, 11, 12 years) analysis of covariance (ANCOVA) with repeated measures on Age. Due to the fact that growth and maturation may have an impact on physical fitness level and its development in youth [24], the following covariates were included in our ANCOVA model: (1) test-specific physical fitness performance at baseline, (2) change in body weight, and (3) change in body height. Changes in body weight and height were calculated as differences between measurements performed at age 9 and 12. When ‘Sex by Age’ interactions reached the level of significance, group-specific post-hoc tests (i.e., paired *t*-tests) were conducted to identify the comparisons that were statistically significant. Additionally, Cohen`s *d* was calculated. According to Cohen [25], 0.20 ≤ *d* ≤ 0.49 indicates a small, 0.50 ≤ *d* ≤ 0.79 indicates a medium, and *d* ≥ 0.80 indicates a large effect size. The significance level was set at *p* < .05. All analyses were performed using Statistical Package for the Social Sciences (SPSS) version 22.0.

Further, normative sex-specific centile values were generated using LMS chartmaker Pro (v2.54, The Institute of Child Health, London) software. More precisely, the Lambda, Mu, and Sigma (LMS) method provided by Cole [26] were applied next to statistical procedures for model fitting and checking [27–29]. For each of the six physical fitness tests, centile curves were calculated which express the distribution of the respective performances as it changes over time. Performance changes were plotted according to age and illustrated using three curves representing the skewness (L for Lambda), the median (M for Mu), and the coefficient of variation (S for Sigma). The skewness expresses the power in the Box-Cox-Transformation which normalizes the data distribution by variance stabilization. Using penalized likelihood with Generalised Akaike Information Criterion (GAIC) the three curves were fitted as cubic splines by nonlinear regression. The extent of curve-smoothing required was expressed in terms of equivalent degrees of freedom (edf) of each L, M, and S curve as measure of its complexity [29]. System´s requirement of curve smoothing included an alternative transformation of original age (o) scale, denoted as rescaled age (r), too. Rescaled age is an empirical transformation based on the shape of the fitted M curve. In a last step, the goodness of model fit was checked by Q-Tests for fit [28] and, if necessary, improved by adjusting edf of L, M, and S curve. All centile-analyses were performed for boys and girls, separately and expressed as tabulated percentiles (P) from P_10_ to P_90_ and as smoothed centile curves showing P_10_, P_50_, and P_90_.

## Results

Anthropometric and physical fitness test data of the study sample sorted by Sex and Age are presented in Table 1. Significant main effects of Age but not of Sex were found for body weight (*p* < .001, *d* = 4.21), body height (*p* = < .001, *d* = 6.88), and BMI (*p* = < .001, *d* = 1.91). More specifically, weight, height, and BMI significantly increased with age in boys as well as in girls. Furthermore, a statistically significant interaction effect of Sex by Age was detected for body height (*F*_[1, 238]_ = 10.3, *p* < .001, *d* = 0.42). Post-hoc analyses indicated a significantly larger somatic growth in girls (9–12 years: *d* = 2.50) than in boys (9–12 years: *d* = 2.03). Moreover, additional statistical analyses were computed between the drop outs (i.e., 60 girls and 46 boys) and the included children and revealed no significant differences in anthropometrics between the two groups.

**Table 1:** Development of anthropometry and physical fitness in 9 to 12 years old boys (n = 152) and girls (n = 88).

Age	9 years		10 years		11 years		12 years		^1^Main/interaction effect: *p*-value (*d*)		
	Boys	Girls	Boys	Girls	Boys	Girls	Boys	Girls	Sex	Age	Sex by Age
body weight (kg)	33.1 (6.1)	31.7 (5.4)	37.3 (7.3)	36.0 (7.0)	42.6 (9.1)	41.0 (8.8)	47.6 (10.0)	46.3 (10.1)	.183 (0.17)	< .001 (4.21)	.873 (0.04)
body height (cm)	140.5 (6.2)	138.1 (5.8)	144.8 (6.7)	142.3 (6.2)	150.8 (7.1)	150.0 (6.9)	156.8 (8.0)	155.9 (7.2)	.066 (0.24)	< .001 (6.88)	< .001 (0.42)
BMI (kg/m^2^)	16.7 (2.5)	16.6 (2.3)	17.7 (2.7)	17.7 (2.7)	18.6 (3.1)	18.1 (3.0)	19.2 (3.1)	18.9 (3.3)	.514 (0.09)	< .001 (1.91)	.101 (0.19)
50-m sprint (s)	9.6 (0.8)	9.8 (0.9)	9.2 (0.9)	9.5 (0.9)	8.9 (0.8)	9.0 (0.8)	8.7 (0.8)	8.8 (0.8)	.314 (0.13)	< .001 (0.69)	.324 (0.14)
ball push (m)	7.91 (1.41)	6.40 (1.30)	8.93 (1.51)	7.56 (1.26)	10.70 (1.92)	8.84 (1.60)	11.57 (2.31)	9.79 (1.71)	< .001 (0.55)	< .001 (0.40)	< .001 (0.36)
triple-hop (m)	7.65 (1.24)	7.26 (1.15)	8.44 (1.19)	8.04 (1.21)	9.17 (1.26)	8.83 (1.16)	9.78 (1.43)	9.37 (1.31)	.096 (0.22)	< .001 (0.67)	.114 (0.19)
stand-and-reach (cm)	96 (8)	100 (8)	96 (8)	101 (7)	95 (9)	103 (7)	96 (9)	104 (8)	< .001 (0.81)	< .001 (0.43)	< .001 (0.61)
star agility run (s)	23.2 (3.3)	23.9 (3.0)	21.0 (2.1)	22.0 (2.1)	19.6 (1.9)	20.3 (1.8)	19.0 (1.9)	19.5 (1.7)	.001 (0.44)	< .001 (1.34)	.004 (0.27)
9-min run (m)	1471 (203)	1319 (204)	1569 (269)	1417 (225)	1606 (248)	1467 (201)	1598 (258)	1469 (218)	.014 (0.32)	< .001 (0.79)	.113 (0.18)

*Notes*. Values are means (± SD)

^1^Results of the Sex by Age ANCOVA (covariate: baseline performance, change in body weight and height) with repeated measures on Age

*d* = effect size Cohen´s *d;*

BMI = Body Mass Index.

### Physical fitness differences by age and sex

Significant main effects of Age were found for all physical fitness tests (all p < .001, d = 0.43–1.34) indicating performance improvements from age 9 to 12. Additionally, main effects of Sex were significant for the ball push test (F_[1, 238]_ = 17.9, p < .001, d = 0.55), the stand-and-reach test (F_[1, 238]_ = 38.7, p < .001, d = 0.81), the star agility run test (F_[1, 238]_ = 11.4, p = .001, d = 0.44), and the 9-min run test (F_[1, 238]_ = 6.1, p = .014, d = 0.32). Boys outperformed girls in the ball push test, the star agility run test, and the 9-min run test, whereas girls achieved better results in the stand-and-reach test. Lastly, statistically significant interaction effects of Sex by Age (adjusted for change in body weight, height, and baseline performance) were detected for the ball push test (*F*_[3, 714]_ = 7.8, *p* < .001, *d* = 0.36), the stand-and-reach test (*F*_[3, 714]_ = 21.9, *p* < .001, *d* = 0.61), and the star agility run test (*F*_[3, 714]_ = 4.4, *p* = .004, *d* = 0.27). Post-hoc analyses regarding performance changes from age 9 to 12 revealed larger improvements in girls than boys for the ball push test (girls: *d* = 2.19; boys: *d* = 1.81), the stand-and-reach test (girls: *d* = 0.55; boys: *d* = 0.07), and the star agility run test (girls: *d* = 1.65; boys: *d* = 1.46). Further, we computed additional statistical analyses between the drop outs (i.e., 60 girls and 46 boys) and the included children and observed no significant differences in physical fitness between the two groups.

### Percentile curves by age and sex

**Fig 3:**
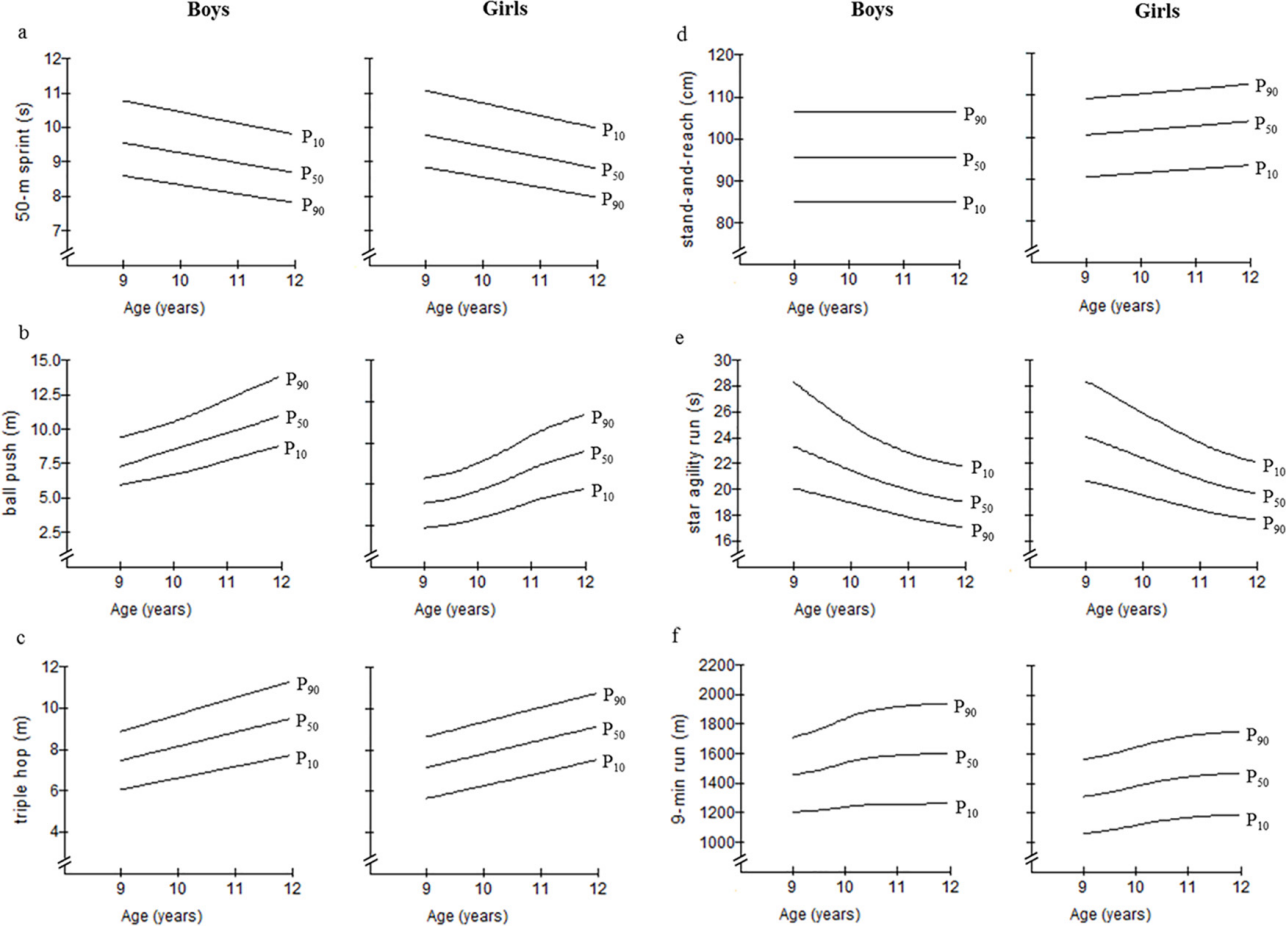
Smoothed LMS curves for the 10th, 50th, and 90th percentiles of the (a) 50-m sprint test, (b) ball push test, (c) triple hop test, (d) stand-and-reach test, (e) star agility run test, and (f) 6-min run test in boys and girls from age 9 to 12 years.

Smoothed age-specific percentiles (i.e., from P_10_ to P_90_) are presented in Table 2 for the 50-m sprint test, the 1-kg ball push test, and the triple hop test. Table 3 illustrates smoothed age-specific percentiles for the stand-and-reach test, the star agility run test, and the 9-min run test. For the same physical fitness tests, smoothed LMS curves for 10^th^, 50^th^, and 90^th^ percentile are depicted in Fig 3. Our data indicate a linear improvement for proxies of speed (both sexes), lower-extremity muscular power (both sexes), and flexibility (girls only). Curvilinear enhancements were found in boys and girls for measures of upper-extremity muscular power, agility, and endurance. For endurance, curvilinear pattern merged in a performance plateau at the age of 12 in both sexes. Notably, no performance development was observed for the stand-and-reach test in boys. Further, margins between P_10_, P_50_, and P_90_ hardly changed over time for the 50-m sprint test in both sexes. The same pattern was found in girls for the triple hop test, the stand-and-reach test, and the 9-min run test. In contrast, margins between percentile curves decreased with advancing age for the star agility run test and increased for the ball push test in both sexes. In addition, an increase of margins between the 10^th^, 50^th^, and 90^th^ percentile was observed for the triple hop test and the 9-min run test in boys.

**Table 2:** Smoothed age- and sex-specific percentile values for the 50-m-sprint (s), ball push test (m), and triple hop test (m).

Age (yrs)	P_10_	P_20_	P_30_	P_40_	P_50_	P_60_	P_70_	P_80_	P_90_
50-m sprint (s)									
Boys ^[1/2/1o]^									
9	10.8	10.3	10.0	9.8	9.5	9.3	9.1	9.0	8.6
10	10.4	10.0	9.7	9.5	9.3	9.1	8.9	8.7	8.3
11	10.1	9.7	9.4	9.2	9.0	8.8	8.6	8.5	8.1
12	9.8	9.4	9.1	8.9	8.7	8.5	8.3	8.2	7.8
Girls ^[1/2/1o]^									
9	11.1	10.6	10.2	10.0	9.8	9.6	9.4	9.1	8.8
10	10.7	10.2	9.9	9.7	9.5	9.3	9.1	8.8	8.5
11	10.3	9.9	9.6	9.3	9.1	8.9	8.7	8.5	8.3
12	10.0	9.5	9.2	9.0	8.8	8.6	8.4	8.2	8.0
ball push (m)									
Boys ^[4/2/1o]^									
9	5.93	6.51	6.67	6.98	7.29	7.63	8.03	8.55	9.39
10	6.67	7.52	7.74	8.13	8.51	8.89	9.31	9.81	10.52
11	7.72	8.63	8.86	9.30	9.73	10.17	10.67	11.27	12.15
12	8.79	9.74	9.99	10.47	10.95	11.45	12.03	12.74	13.83
Girls ^[0/4/1r]^									
9	4.85	5.37	5.74	6.06	6.35	6.65	6.97	7.34	7.86
10	5.42	5.99	6.41	6.76	7.09	7.42	7.78	8.19	8.77
11	6.45	7.13	7.63	8.05	8.44	8.84	9.26	9.75	10.44
12	7.23	7.99	8.55	9.02	9.46	9.91	10.38	10.93	11.70
triple hop (m)									
Boys ^[0/2/1o]^									
9	6.06	6.73	6.89	7.19	7.47	7.75	8.04	8.39	8.88
10	6.61	7.34	7.52	7.84	8.15	8.45	8.78	9.16	9.69
11	7.16	7.95	8.15	8.50	8.83	9.16	9.51	9.92	10.49
12	7.71	8.56	8.77	9.15	9.51	9.86	10.24	10.69	11.30
Girls ^[0/2/2o]^									
9	5.65	6.16	6.53	6.85	7.14	7.44	7.75	8.12	8.63
10	6.26	6.79	7.17	7.50	7.81	8.11	8.44	8.82	9.36
11	6.89	7.43	7.82	8.16	8.47	8.79	9.12	9.52	10.06
12	7.53	8.08	8.48	8.82	9.14	9.46	9.80	10.20	10.75

*Notes*. P = percentile; in square parentheses: equivalent degrees of freedom (edf) for the chosen model of L/M/S method; L = skew; M = median; S = coefficient of variation; o = original age; r = rescaled age.

**Table 3:** Smoothed age- and sex-specific percentile values for the stand-and-reach test (cm), star agility run test (s), and 9-min run test (m).

Age (yrs)	P_10_	P_20_	P_30_	P_40_	P_50_	P_60_	P_70_	P_80_	P_90_
stand-and-reach (cm)									
Boys ^[1/2/1o]^									
9	85	90	91	93	96	98	100	103	106
10	85	90	91	93	96	98	100	103	106
11	85	90	91	93	96	98	100	103	106
12	85	90	91	93	96	98	100	103	106
Girls ^[1/2/1o]^									
9	91	94	97	99	101	102	104	106	109
10	91	95	98	100	102	103	105	108	110
11	92	96	99	101	103	105	106	109	112
12	93	97	100	102	104	106	108	110	113
star agility run (s)									
Boys ^[1/3/3r]^									
9	28.3	26.3	25.0	24.1	23.3	22.5	21.8	21.4	20.1
10	25.0	23.6	22.7	22.0	21.4	20.9	20.3	20.0	19.0
11	22.8	21.7	21.0	20.4	19.9	19.5	19.0	18.8	17.8
12	21.7	20.7	20.0	19.5	19.0	18.6	18.1	17.9	17.0
Girls ^[1/3/2r]^									
9	28.3	26.8	25.7	24.8	24.1	23.3	22.6	21.7	20.7
10	25.9	24.6	23.7	23.0	22.4	21.8	21.2	20.5	19.5
11	23.6	22.6	21.9	21.3	20.8	20.3	19.7	19.2	18.4
12	22.1	21.2	20.6	20.1	19.7	19.3	18.8	18.3	17.6
9-min run (m)									
Boys ^[0/2/1r]^									
9	1205	1324	1354	1407	1457	1507	1561	1623	1710
10	1240	1381	1416	1479	1538	1597	1660	1734	1836
11	1259	1415	1454	1523	1588	1654	1723	1805	1918
12	1261	1421	1461	1533	1600	1666	1738	1822	1938
Girls ^[0/3/1r]^									
9	1061	1147	1209	1262	1312	1362	1415	1477	1563
10	1117	1208	1273	1329	1381	1434	1490	1555	1646
11	1170	1265	1333	1392	1447	1501	1560	1629	1724
12	1189	1286	1355	1415	1471	1526	1586	1655	1752

*Notes*. P = percentile; in square parentheses: equivalent degrees of freedom (edf) for the chosen model of L/M/S method; L = skew; M = median; S = coefficient of variation; o = original age; r = rescaled age.

## Discussion

The present study provides longitudinal data on age- and sex-specific physical fitness percentiles in healthy children aged 9–12 years. The major strength of the present longitudinal study as compared to cross-sectional studies is that individual changes in timing and tempo of growth are taken into account.

### Age and sex differences in physical fitness

Initially, we hypothesized that physical fitness levels increase from age 9–12. Our findings confirm the first hypothesis because physical fitness significantly improved over the four year study period in males and females (except for flexibility in boys). Data from cross-sectional studies confirm our findings in as much as physical fitness enhancements were reported in groups of increasing age [7, 9, 30]. For example, Woll et al. [30] demonstrated higher performance levels (i.e., endurance, static/dynamic balance, lower-body muscular power, muscle endurance, coordination under time pressure) with age in 4- to 17-years-olds. Similar results are reported by Catley and Tomkinson [9] who analyzed performances in health-related fitness tests (i.e., 1.6-km run, 20-m shuttle run, 50-m sprint, basketball throw, standing broad jump, push-ups, sit-ups, hand-grip strength) in 9- to 17-years-olds from 1985 up to 2009. Age-related differences in physical fitness are typically attributed to growth (i.e., increase in body size, body weight, and body dimensions) and maturation (i.e., somatic, skeletal, and sexual maturity) that occur during childhood and adolescence [24]. Furthermore, no statistically significant improvements were detected for the stand-and-reach test in boys. This is in line with a cross-sectional study conducted by Castro-Pinero et al. [31] who tested flexibility in 6- to 17-years-olds. As a result, sit-and-reach test performance did not significantly improve in boys [31]. Amongst other reasons, this can most likely be explained by maturational processes of joint structures and by an increase in muscle mass, particularly in boys [24].

Our study findings also confirm our second hypothesis in as much as we observed significant sex differences in physical fitness development over time. In fact, our results revealed that boys outperformed girls in the ball push test (medium effect size), the star agility run test (small effect size), and the 9-min run test (small effect size). In contrast, girls achieved higher values in the stand-and-reach test (large effect size). These results are in line with findings from cross-sectional studies [6, 12, 30, 32]. In fact, Castro-Pinero et al. [6] reported that boys aged 8–9, 10–11, and 12–13 achieved significantly better performances in the ball throw test compared to girls of the same age. The observed sex differences can most likely be attributed to a better ratio of strength relative to body weight in boys compared to girls, particularly in the upper limbs and trunk [33]. In addition, relative muscle strength of the upper limbs (i.e., normalized to muscle cross-sectional area) is already higher in boys as compared to girls during childhood [34]. Further, Roriz De Oliveira et al. [12] found significantly better agility (i.e., 4 x 10-m shuttle run test) in 6- to 10-year-old boys compared to girls. The better agility performance in boys as in girls can be explained by their higher absolute and relative (i.e., in relation to body weight and fat-free mass) anaerobic power values obtained during the 30-s Wingate Anaerobic Test [35]. In addition, De Miguel-Etayo and colleagues [32] reported significantly better endurance (i.e., 20-m shuttle run test) in 6- to 9-years-old boys as compared to girls. The observed sex differences in endurance might be explained by a higher maximal aerobic capacity in boys. For example, longitudinal analyses in children aged 8–18 revealed that before the age of 10–12 years, girls`average VO_2_max reaches about 85–90% of that of boys. Likewise, sex differences exist when body weight is taken into account (90–95% of male mean values) [24]. Finally, higher flexibility scores (i.e., stand-and-reach test, back-saver sit-and-reach test) were reported for girls as compared to boys [30, 32]. This might be explained by a higher percentage of body fat and a lower percentage of muscle mass due to higher circulating levels of estrogens or lower circulating levels of androgens in girls compared to boys. As a result, tissue density is lower in girls which may result in better flexibility [24, 36]. Besides these physiological factors, behavioral aspects may also account for the observed sex differences in flexibility during childhood. For example, Haywood and Getchell [37] argued that stretching exercises are socially more acceptable for girls than vigorous (muscle strengthening) exercises and that a higher proportion of girls participates in gymnastics and dance as compared to boys.

In the present study, the observed improvements in physical fitness with advancing age occurred at different rates which are indicated by linear versus curvilinear percentile models. More specifically, linear improvements were found for proxies of speed (both sexes), lower-extremity muscular power (both sexes), and flexibility (in girls). In contrast, curvilinear models are effective for both sexes regarding measures of upper-extremity muscular power, agility, and endurance. Moreover, our adjusted analyses (i.e., changes in body weight, height, and baseline performance) revealed significant Sex by Age interaction effects. These were found for the ball push test, the stand-and-reach test, and the star agility run test. Post-hoc analyses revealed that performance progression for the ball push test and the star agility run test was slightly larger in girls compared to boys but reached similar effect sizes (i.e., large effect). With regards to the stand-and-reach test, performance development was substantially faster in girls than in boys as indicated by a medium vs. small effect size, respectively. These findings may indicate a timed and capacity-specific development pattern during childhood as described by the biological concept of “critical or sensitive maturational periods” [38]. Critical periods can be characterized as periods during which ontogenetic development reaches a qualitatively new level that provides opportunities for the further improvement of an organ, tissue, and/or physiological functions [38]. Sex-specific critical periods of accelerated (i.e., curvilinear) performance improvements have been reported for several proxies of physical fitness (except agility) [38–40]. Furthermore, accelerated gains in muscle strength were detected for different ages depending on the investigated strength capacity (e.g., maximal voluntary isometric strength, muscular power). More specifically, intense improvements in muscular power (boys: 7–9 years and 13–16 years; girls: 6–8 years and 11–12 years) occur earlier than those in maximal voluntary isometric strength (boys: 14–16 years; girls: 12–13 years) [39]. With respect to upper-extremity muscular power (i.e., ball push test), we found the highest annual gains between the ages of 9–11 years in girls (17–18%) and 10–11 years in boys (20%). The present study provides new in-depth insight in the development of agility. Accelerated improvements were found for the star agility run test in girls aged 9–11 (8%) and boys aged 9–10 (10%). In addition, we observed an accelerated improvement in aerobic endurance at the age of 9–10 in girls (7%) and boys (7%). This is in line with previous results of Andersen and colleagues who assessed aerobic endurance in a longitudinal approach in 8- to 12-year-old children [13]. As a result the largest performance gains (8–10%) were reported for girls and boys aged 9–10 years.

Of note, children’s performance development in physical fitness is affected by somatic growth. Sex-specific changes in parameters like body height and weight particularly occur from age 9 to 12 [24]. More specifically, a significantly larger growth in body height has been reported for girls as indicated in growth velocity curves [24]. For example, Tanner et al. [41] showed an earlier beginning of an accelerated height gain (i.e., cm/year) in girls (10 years) as compared to boys (12 years). Our data is in accordance with the literature because the statistical analyses revealed a significantly larger growth of body height in girls compared to boys. This may in fact explain the observed faster performance development in the ball push test, the star agility run test, and the stand-and-reach test in girls.

### Physical fitness percentiles

The present study reports physical fitness percentiles from longitudinal data. More specifically, the present study provided age- and sex-specific percentile values obtained from the same children over a four year study period (i.e., from 9–12 years of age). Given that there is no longitudinal study available in the literature that examined physical fitness percentiles in children, the present findings have to be compared with results from cross-sectional studies. We applied six physical fitness tests in the present study. Of those, three (i.e., stand-and-reach test, 9-min run test, 50-m sprint test) were frequently reported in other studies which is why our data was primarily compared to the findings from these studies. With regards to the stand-and-reach test, Bös et al. [15], reported percentile values for German boys (n = 150 to 158) and girls (n = 141 to 154). A difference between Bös et al. [15] and our findings was observed in as much as the boys investigated study were more flexible (between 1–4 cm) in Bös et al. [15] compared to ours. This finding was irrespective of the age group (9, 10, 11, 12 years of age) considered. For girls, age-specific differences between the two studies were found also. Bös et al. [15] reported values that were slightly (i.e., 1–2 cm) better than ours for 9- to 10-years-olds. However, our data set indicated better performances for 11- to 12-year-olds. Regarding the 9-min run test, Bergmann et al. [42] conducted a study in Brazil with 7- to 12-year old students and reported age- and sex-specific mean values. Irrespective of the examined age category, children achieved shorter distances (boys: 173–361 m, girls: 216–261 m) when compared to our study sample. Lastly, Catley and Tomkinson [9] summarized normative fitness data of 9-17-year-old Australians from 15 studies (sample size range: n = 54–2,612). Irrespective of sex and age (9–12 years), a comparison between the study of Catley and Tomkinson [9] and our study revealed that the reported values for the 50-m sprint were approximately two seconds slower for the 10^th^, 20^th^, and the 30^th^ percentile; nearly the same for the 40^th^ and the 50^th^ percentile; and 1–2 seconds faster for the 50^th^ to the 90^th^ percentile. Several reasons might be responsible for the observed differences in percentile values between the present findings and the aforementioned studies. For example, a longitudinal study design was used in the present study, whereas a cross-sectional approach was applied in the other studies. Cross-sectional studies are limited because they do not control for individual changes in timing and tempo of physical fitness development. Further, differences in the applied methodological approach may have had an impact as well. In fact, we randomly selected children from rural and urban areas. Of note, none of the aforementioned studies provided information regarding their sampling procedure. Thus, urban or rural areas may have been over- or under-represented in those studies as compared to our study. In this regard, it has been reported that physical fitness and its development differs between rural and urban children, with better values and larger improvements for children living in urban compared to rural areas [43, 44].

The reported percentile values are of particular importance for professionals (i.e., teachers, coaches, and fitness instructors) working in educational (e.g., schools), athletic (e.g., talent identification), and health-care settings (e.g., sports/fitness clubs). More specifically, data from this longitudinal study can be used to grade motor performance and motor performance development of children aged 9–12 during physical education. With regard to long-term athlete development and the identification of talents, the age range of 9–12 years is an important stage (i.e., “Learn to Train stage”) during which children are supposed to take up general sporting skills that build a strong foundation for subsequent stages during long-term athlete development (i.e., “Train to Train”, “Train to Compete”, and “Train to Win” stage) [45]. Our findings of percentile values may help to detect high fit children at a certain point and/or over time. More specifically, children performing above the 80^th^ percentile can be classified as ‘very high fit’. Sports organizations/associations and sports clubs could offer multifaceted and appealing sport programs to the previously identified children that further their athletic development to finally become a high performance athlete.

The identification of low performing children is as important as the identification of youth athletes because there is evidence of an association between low scores of cardiorespiratory fitness, muscle strength as well as overall fitness and cardiovascular risk [46–48]. Thus, children with values below the 20^th^ percentile should be targeted and introduced to fitness promoting programs. These initial low performers can be followed longitudinally to see if their motor performance improves over time and if the respective fitness promoting program is successful.

### Limitations and strengths of the present study

This study has some limitations and strengths, which warrant discussion. Our findings are limited to healthy boys and girls aged 9 to 12 years. Further, we did not assess additional factors such as biological maturation (e.g., Tanner stages), physical activity (e.g., amount or intensity of being physically active), media use (e.g., time spent watching TV or playing computer games), and socioeconomic status (e.g., parental education, occupation, income status) in our analyses that may have an impact on children’s physical fitness level and development. Besides these limitations, some strengths should also be addressed. First, a longitudinal approach was used that allows us to deduce effective physical fitness development in children over time (i.e., individual changes in timing and tempo). Second, a relatively large sample of 240 boys and girls was included. Third, findings from a large number of physical fitness tests were reported that are part of frequently used and published fitness test batteries in different countries (e.g., Brazil, Australia). Thus, we were able to compare data from our German sample with international data (i.e., stand-and-reach test, 50-m sprint test, 9-min run test). Forth, the applied field tests represent health- (cardiorespiratory endurance: 9-min run test, flexibility: stand-and reach test) and skill-related (agility: star run test, speed: 50-m sprint test, power: 1-kg ball push test, triple hop test) components of physical fitness in youth [17]. In children, the assessment of health-related physical fitness is important because cardiorespiratory endurance for instance tracks from childhood over adolescence into adulthood and thus predicts physical fitness later on in life [49, 50]. Further, skill-related physical fitness is an important predictor of children’s physical activity level. It has been shown that 6-year-old children with a high motor skill level exhibit a significantly higher physical activity level over the next three years compared with children with low or middle motor skill levels [51]. Finally, field tests are easy to administer and require little equipment and personnel compared to laboratory-based tests. Notably, a relatively large amount of students can be tested in a short period of time.

## Conclusions

This longitudinal study produced age- and sex-specific physical fitness percentiles for six different tests in healthy children aged 9–12. In girls as compared to boys, physical fitness development was slightly faster for upper-extremity muscular power and agility but substantially faster for flexibility. Furthermore, accelerated (curvilinear) improvements were observed for upper-extremity muscular power (boys: 10–11 years; girls: 9–11 years), agility (boys: 9–10 years; girls: 9–11 years), and endurance (boys: 9–10 years; girls: 9–10 years) indicating a timed and capacity-specific physical fitness development during childhood. As a consequence, sex-specific growth and maturational processes already have an impact on physical fitness development in 9- to 12-year-olds. Most important, teachers, coaches, and fitness instructors can use the obtained percentile values as approximate benchmarks to identify children with specific fitness characteristics. More specifically, percentiles can be introduced to educational settings (e.g., age- and sex-specific grading of motor performances) as well as to programs of long-term athlete development (i.e., high achievers) and/or to promote health-related physical fitness in low achievers.
